# Multifocal Medulloblastoma in an Adult Patient: Description of a Rare Presentation and Review of the Literature

**DOI:** 10.1155/2020/4502878

**Published:** 2020-09-12

**Authors:** Irene Troncon, Angela Guerriero, Sabrina Rossi, Monica Ronzon, Marta Padovan, Caccese Mario, Lucia Zanatta, Luisa Toffolatti, Elisabetta Marton, Giuseppe Lombardi, Angelo Paolo Dei Tos, Giuseppe Canova

**Affiliations:** ^1^Department of Neurosurgery, Santa Maria Della Misericordia Hospital, Rovigo, Italy; ^2^Department of Pathology and Molecular Genetics, Treviso General Hospital, Treviso, Italy; ^3^Department of Pathology, Bambino Gesù Children's Hospital, Rome, Italy; ^4^Department of Neuroradiology, Treviso Regional Hospital, Treviso, Italy; ^5^Department of Oncology, Oncology 1, Veneto Institute of Oncology IOV-IRCCS, Padova, Italy; ^6^Department of Neurologic Surgery, Treviso Regional Hospital, Treviso, Italy; ^7^Department of Medicine, University of Padova School of Medicine, Padova, Italy

## Abstract

Medulloblastoma is an embryonal neuroepithelial tumor that affects mainly childhood and more rarely adults. Medulloblastoma occurring as multiple nodules at diagnosis is a rare and tricky presentation. Here, we describe the case of a previously healthy 47-year-old woman with multiple posterior fossa cerebellar tumors. Histological, immunohistochemical, and molecular analyses were performed to best characterize the two excised lesions. The histopathological analysis revealed different variants of medulloblastoma in the excised nodules, one being extensive nodularity, rare in adults, and the other desmoplastic/nodular with areas of anaplasia. Immunostains and molecular analysis classified both nodules as SHH medulloblastoma. Adult medulloblastoma is extremely rare. Important differences exist between adult medulloblastoma and medulloblastoma arising in children and infants. Such differences are in location, distribution of histological variants and of molecular subgroups, survival rates, and therapeutic options. An extensive morphological and molecular characterization of such rare tumors is necessary to choice the best-tailored therapy.

## 1. Introduction

Medulloblastoma (MB) is an embryonal neuroepithelial tumor arising in the cerebellum or dorsal brain stem presenting mainly in childhood and consisting of densely packed small, undifferentiated cells with mild to moderate nuclear pleomorphism and high mitotic count [1].

MB is the most common malignant central nervous system neoplasm in childhood representing 25% of all pediatric brain tumors and 30-40% of primary posterior fossa tumors [2]. On the contrary, it is very rare in adults accounting for <1% of intracranial tumors in patient aged >16 [3]. Based on morphology, MB was classified as classic, desmoplastic/nodular, with extensive nodularity (MBEN), and large cell/anaplastic (LCA).

In 2012, an international consensus on MB subgroups was reached amongst the pediatric neurooncology community reporting four distinct MB subgroups: WNT, SHH, Group 3, and Group 4 [4]. Tumors of the first two subgroups show activation of the WNT and SHH cell signaling pathways, respectively [4]. Groups 3 and 4 comprise all MB lacking WNT and SHH activation. Recently, an international meta-analysis has clarified the complexity and heterogeneity of Groups 3 and 4 MB identifying eight biologically and clinically relevant subtypes [5].

Typically, MB presents as a solitary mass in the fourth ventricle or in the cerebellar parenchyma; multifocal cases are rare in patients not affected by familial tumor syndromes, and only six cases have been described in adults to date [6–11]. We describe the case of an adult patient presenting with cerebellar multifocal MB; detailed morphological, molecular, and cytogenetic features were reported.

## 2. Case Presentation

### 2.1. Clinical History

**Figure 1:**
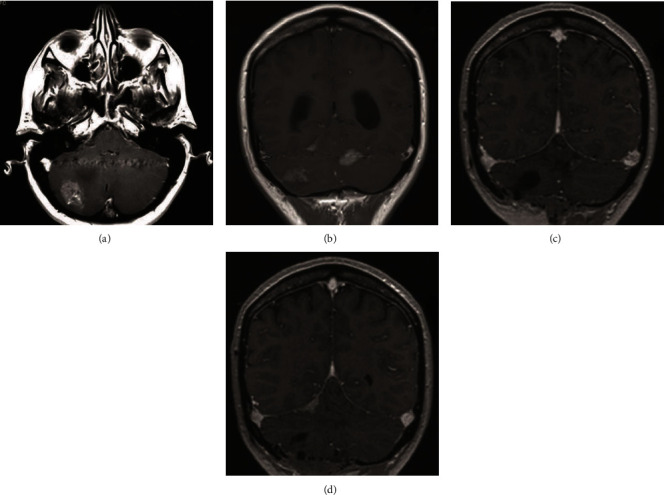
RMI T1-weighted with gadolinium. Preoperative exam shows lesions with perilesional edema, some cystic aspect and heterogeneous contrast enhancement (a, b). Postoperative exam shows as the two major lesions were excised and the small enhancing subtentorial paravermian right spot was evident (c, d).

A 47-year-old woman presented with a one-month history of headache and vomiting. Her personal and family history was irrelevant. A CT scan showed multiple posterior fossa cerebellar lesions associated with perilesional edema and triventicular hydrocephalus. An MRI study confirmed the presence of a major lesion localized in the right cerebellar hemisphere, another lesion was in the left paravermian hemisphere, and a small enhancing spot was subtentorial paravermian right localized. Lesions appeared with some cystic aspects, perilesional edema, and intense and heterogeneous contrast enhancement (Figures 1(a) and 1(b)). Cerebellar tonsils appeared herniated caudally, the fourth ventricle was flattened, and triventricular hydrocephalus was present with signs of exudation.

Total-body imaging and hematological evaluations excluded other primary tumors. At first, the patient underwent a ventricular peritoneal shunting with a Codman Hakim programmable valve (120 mm H_2_O), without complications. Afterwards, she underwent median suboccipital craniotomy to remove the right cerebellar hemispherical lesion and the paravermian left lesion (Figures 1(c) and 1(d)).

A spinal MRI performed 10 days after surgery excluded spinal tumor dissemination. In the postoperative course, the patient presented no complications. The adjuvant treatment provided craniospinal irradiation (36 Gy in 20 fractions) followed by six cycles of chemotherapy: intravenous cisplatin (75 mg/m^2^) and oral lomustine (75 mg/m^2^) on the 1st day and intravenous vincristine (1.5 mg/m^2^) on the 1st, 8th, and 15th day. From the 2nd cycle, the dose was reduced to 75% due to neutropenia, and vincristine was not administered in the last cycle. Follow-up MRI evaluation performed every 2 cycles did not show progression at 30 months after diagnosis.

### 2.2. Pathological and Molecular Findings

**Figure 2:**
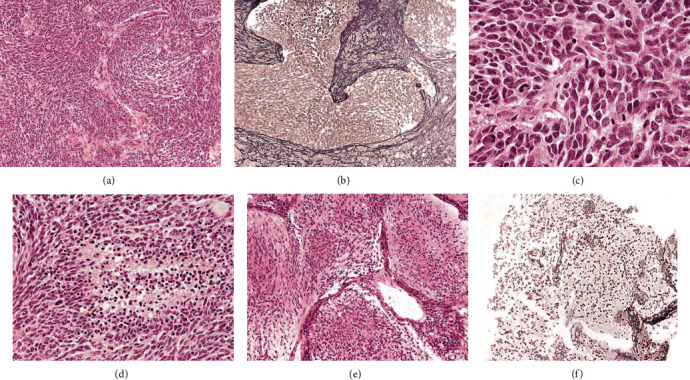
Histological aspects of the two excised lesions. (a) The right hemispherical lesion made of highly cellular nodules composed of fusiform hyperchromatic cells set in an intervening highly cellular background (haematoxylin and eosin stain, HE; original magnification x10). (b) Nodules are reticulin-free; internodular areas are reticulin-rich (reticulin stain, original magnification x10). (c, d) Areas of anaplasia with highly pleomorphic cells with numerous mitosis and foci of necrosis are present (haematoxylin and eosin stain, HE; original magnification x40 (c), x20 (d)). (e) The paravermian left lesion consisted of expanded nodules made of small cells (haematoxylin and eosin stain, HE; original magnification x10). (f) Expanded nodules are reticulin-free, surrounded by reticulin-rich interlobular areas (reticulin stain, original magnification x10).

Multiple grayish fragments were examined for the two lesions. The right hemispherical lesion consisted of highly cellular reticulin-free nodules made of fusiform hyperchromatic cells set in an intervening highly cellular, reticulin-rich background (Figures 2(a) and 2(b)). Areas of anaplasia with highly pleomorphic cells, numerous mitotic, and apoptotic figures and foci of necrosis were present (Figures 2(c) and 2(d)).

The paravermian left lesion consisted of expanded reticulin-free nodules made of small cells with round nuclei surrounded by densely packed reticulin-rich cells in the interlobular areas (Figures 2(e) and 2(f)).

**Figure 3:**
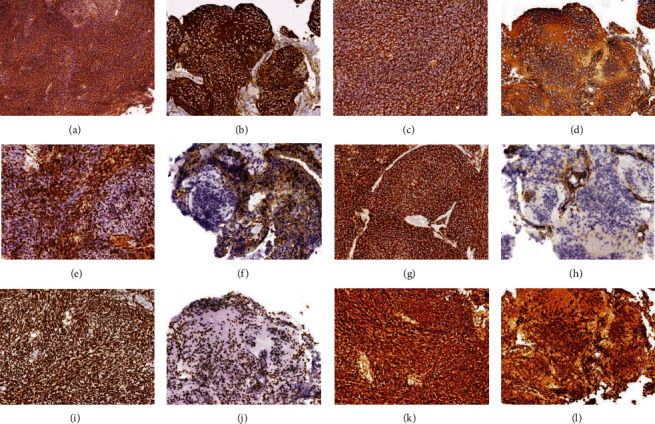
Immunohistochemical panels of the two excised lesions. (a, b) Tumors are strongly positive for synaptofisin (original magnification x4 (a), x10 (b)). (c, d) Beta-catenin has a cytoplasmic distribution (original magnification x10). (e, f) YAP1 is diffusely positive (original magnification x10). (g, h) GAB1 is positive and diffuse in the right cerebellar lesion and predominantly internodular in the left lesion (original magnification x10). (i, j) Tumor cells maintain the expression of INI-1 (original magnification x10). (k, l) Tumor cells maintain the expression of BRG1 (original magnification x10).

Immunohistochemistry showed the tumor cells of both lesions to be strongly positive for synaptofisin (Figures 3(a) and 3(b)) and for Neu-n in the nodular areas. Beta-catenin showed no nuclear staining (Figures 3(c) and 3(d)). Tumor cells showed nuclear and cytoplasmic positivity for YAP1 (Figures 3(e) and 3(f)) and for GAB1, which was diffused in the right lesion and with an internodular distribution in the left lesion (Figures 3(g) and 3(h)). Olig2 and GFAP were negative in tumor cells and positive in rare intermixed glial cells, INI1, and BRG1 were retained (Figures 3(i)–3(l)). P53 was expressed in 5% of neoplastic cells. The proliferative index (Ki67) was 40% in anaplastic areas of the right hemispherical lesion and 30% in the internodular areas of the left lesion.

Based on morphological and immunohistochemical results, the right lesion was diagnosed as desmoplastic/nodular MB with severe anaplasia, SHH-activated *TP53-*wildtype; the left paravermian lesion was diagnosed as MB with extensive nodularity, SHH-activated *TP53-*wildtype.

The presence of *TERT* promoter mutation and the absence of *TP53* mutations supported the diagnosis.

CGH microarray performed on the right hemispherical lesion evidenced monosomy of chromosomes 1, 9, 10, 11, 14, 15, 16, 17, 19, and 22 and a deletion of 52 Mb of the short arm of chromosome x.

## 3. Discussion

Adult MB is very rare with an annual incidence ranging from 0.5 to 20/1 million according to various reports [3, 12]. Histological variant and molecular subgroup prevalence varies among age groups.

In adults, SHH MB forms the largest group accounting for 57% of all tumors, followed by Group 4 (28%), WNT (13%), and Group 3 (2%) [13]. The SHH subgroup is the most frequent also in infants (<3 years); WNT and Group 4 are more frequently seen in children (3-16 years) [4]. Histologically, the desmoplastic/nodular variant is the most frequent in adults and infants; the classic variant is the most frequent in children [4].

Differences exist in survival rates among age groups with the exception of SHH tumors, which have a similar 5-year survival rate of 70% in all age groups [4, 13]. WNT and Group 4 have the worst prognosis in adults; Group 3 have a poor prognosis in all age groups with the shorter overall survival in adult patients (OS: 25%) [4].

Chang's classification divides all age-group MB patients in average and high risk. Average-risk patients present neither metastases nor residual disease after surgery (residual disease defined as >1.5cm^2^) and have favorable histology (not large cells/anaplastic histology). High-risk patients have metastases and/or residual disease > 1.5cm^2^ and/or large cells/anaplastic histology [14].

For all patients with MB, the first treatment is surgery and the most radical excision should be undertaken [15]. Since surgery alone is associated with a high incidence of recurrence, complementary therapy is needed [16]. Adjuvant radiotherapy is the cornerstone in the treatment of MB. Adults with average-risk MB are treated with postoperative radiation alone, as chemotherapy role is still controversial. In pediatric patients, reduced-dose craniospinal irradiation therapy plus a boost to the posterior fossa with concomitant chemotherapy obtained encouraging results [13, 17]. High-risk patients receive craniospinal irradiation; there is no consensus on chemotherapy in either pediatric or adult settings [13]. In our case, the presence of anaplasia in the right hemispherical lesion induced clinicians to add adjuvant chemotherapy.

Synchronous multifocal MB is rare with only six cases previously described in adults (Table 1) [6, 11]. Histological and molecular data were available in a minority of cases [9–11].

Our case, unlike the previous ones, shows different histology in the excised nodules. However, both lesions were SHH MB, *TP53*-wildtype. CGH microarray performed on the lesion with areas of anaplasia showed absence of monosomy of chromosome 6, no amplification of *N-MYC* and *C-MYC* genes, and absence of isochromosome 17q, excluding Group 3 and Group 4 despite the presence of areas of anaplasia. CGH array showed the monosomy of chromosome 9, which could indicate PTC1 deletion.

PTC1 is an inhibitor of hedgehog signaling, important in cerebellar development. *PTCH1* tumor suppressor gene is located on chromosome 9q22.3; loss of heterozygosity (LOH) in this region has been demonstrated in many sporadic desmoplastic/nodular MB [18]. Activation of the pathway occurs when the ligand SHH binds to PTC1, releasing it from SMO inhibition and activating GLI transcription factor [19]. In the last years, target therapy with inhibitors of SMO receptor has emerged showing efficacy in SHH MB with increasing activity of SMO factor [20]. However, at date, all these target inhibitors are not yet approved in the therapy of MB.

Presentation of MB in multiple nodules is rarely seen. MB tends to spread through the cerebrospinal fluid way with dissemination among the neuraxis [1]. Posterior fossa is the most common site of relapse. In adults, recurrences occur late (>4 years after treatment) in contrast with pediatric population in which 75% of recurrences occur in the first two years of treatment [2, 12, 21].

In cases with multifocal presentation, efforts need to define such lesions as metastasis or as synchronous independent MB, important for the definition of the risk class and for therapeutic choices.

In our case, both lesions rely on the activation of the SHH pathway but whether the two lesions share the same molecular trigger or different members of the SHH pathway are altered, could not be assessed. The different histology could be suggestive of independent synchronous MB but it could be confirmed only performing a complete analysis of their molecular profiles. Moreover, it is essential to consider intratumor heterogeneity. In fact, it has been ascertained that cells of SHH MB arise from the granule cell precursors (GPCs), but can show transcriptionally different stage of GCP differentiation [22]. Therefore, it is difficult decipher the tumor biology and develop tumor diagnostics based on the tumor bulk [22].

In conclusion, on the basis of such novel knowledge, we think that it is essential to conduct an in-depth analysis that involves the use of multiple molecular and cytogenetic techniques to complete and enrich the histological diagnosis also in order to define the prognostic behavior and any target therapies of such rare tumors.

## Figures and Tables

**Table 1:** Clinical, histological, and molecular features of multifocal medulloblastomas [6–11].

Author, year	Age, gender	Symptoms	Location	N. of lesions	Histology	Molecular subgroup
Shen, 1988	31, male	Headache, vomiting	Cerebellar hemispheres	3	Us	Us
Spagnoli, 1990	36, female	Headache, gait disturbance, intention tremor, nistagmus	Cerebellar hemispheres	3	Us	Us
Gliemroth, 1998	54, male	Headache, nausea, vomiting, bilateral dysmetria, gait disturbance	Cerebellar hemispheres, left occipital lobe	2	Us	Us
Ciccarino, 2012	31, male	Headache, ataxia	Cerebellar vermis, cerebellopontine angle	2	Desmoplastic/nodular	Us
Balik, 2015	39, female	Headache	Cerebellar vermis, left hemisphere	3	Classic	SHH
Saad, 2017	41, male	Headache, vertigo	Cerebellar vermis and hemispheres, bilateral caudate nuclei, and left temporale lobe	5	Classic	SHH
Our case	47, female	Headache, vomiting	Cerebellar vermis, cerebellar hemispheres	3	Desmoplastic/nodular, extensive nodularity	SHH

Legend: Us: unspecified.
